# Primary cilia as a molecular convergence hub linking neurodevelopment and autism spectrum disorder

**DOI:** 10.3389/fncel.2026.1914796

**Published:** 2026-09-22

**Authors:** Arianna Boscato, Lorenzo Milesi, Marta Biagioli, Alice Tata

**Affiliations:** NeuroEpigenetics Laboratory, Department of Cellular, Computational and Integrative Biology (CIBIO), University of Trento, Trento, Italy

**Keywords:** autism spectrum disorder, ciliopathies, gene network analysis, neurodevelopment, primary cilia

## Abstract

The primary cilium, a highly conserved microtubule-based organelle, orchestrates a broad repertoire of signal transduction cascades required for embryonic development, tissue homeostasis and cellular communication. Disruption of ciliary structure or function results in a heterogeneous group of multisystem disorders known as ciliopathies. Emerging genomic and transcriptomic evidence indicates that perturbation of ciliary architecture or signaling may also contribute to neurodevelopmental conditions and specifically to autism spectrum disorder (ASD). Genes linked to ASD show a wide range of genetic and phenotypic characteristics: spanning from syndromic neurodevelopmental disorders with overlapping ciliopathy features to idiopathic ASD. Yet the mechanistic contribution of ASD risk genes to ciliary biology remains insufficiently characterized. This study aims to explore the molecular convergence between ciliopathies and ASD by systematically interrogating ciliary gene datasets (CiliaCarta and SysCilia GSv2) in cross-comparison to ASD-associated gene repositories (SFARI Gene). This integrative analysis identifies 105 high-confidence shared genes, which segregate into four mechanistic modules: (i) structural determinants of cilium assembly and maintenance, (ii) components of cilium-dependent signaling pathways, (iii) regulators of cilium-associated gene expression and (iv) cilium associated pathways. We propose that ciliary dysfunction constitutes a convergent mechanism, perturbing neurodevelopmental signaling, neuronal polarity and synaptic maturation in a subset of ASD cases. Clarifying the role of the genes shared between ciliogenesis and neurodevelopmental signaling will refine genotype-to-phenotype correlations, expand the spectrum of cilia-associated neurodevelopmental disorders and potentially uncover therapeutically actionable hubs within cilium-dependent signaling circuitry.

## 1 Introduction

Autism spectrum disorder (ASD) is a heterogeneous and highly heritable neurodevelopmental condition, often diagnosed within the first 3 years of life. Individuals exhibit impaired social interaction and communication, as well as repetitive behaviors and narrowed interests. Those features may be associated with other major co-occurring comorbidities including epilepsy, sensory abnormalities, attention deficits and depression; which can further impact on ASD patients’ overall quality of life (Devlin and Scherer, 2012; Lai et al., 2014; Pinto et al., 2014; De Rubeis and Buxbaum, 2015). Some cases of ASD occur within syndromic disorders such as: Tuberous Sclerosis, Joubert syndrome, Rett syndrome and Fragile X syndrome, which are defined by genetic, systemic and neurobehavioral characteristics (Carroll et al., 2021; Karalis et al., 2022; Kereszturi, 2023). ASD is characterized by great genetic heterogeneity: although most of the known high-confidence ASD risk genes harbor *de novo* dominant disruptive pathogenic variants, affecting one allele and resulting in haploinsufficiency, missense and polygenic contributions also play a significant role (Iossifov et al., 2012; O’Roak et al., 2012; Sanders et al., 2012; Pinto et al., 2014; De Rubeis and Buxbaum, 2015; Fu et al., 2022; Rolland et al., 2023). Widespread evidence links ASD to brain connectivity defects, with synaptic dysfunction, dysregulation of axonal growth and pathfinding and disruption of excitatory-inhibitory neurons balance (Lalli et al., 2020; Carroll et al., 2021). Our perspective mini-review explores whether these complex macroscopic and microscopic neurodevelopmental defects could be linked to dysregulated primary cilia (PC) signaling and functionality during brain development as previously inferred by different studies (Youn and Han, 2018; Andreu-Cervera et al., 2021; Hasenpusch-Theil and Theil, 2021; Stoufflet and Caillé, 2022; Zaidi et al., 2022). PC are sensory organelles assembled in almost all quiescent cells (G_0_) from the centrosome and disassembled when cells re-enter cell cycle. During this process, the mother centriole docks onto the plasma membrane and elongates to form the microtubule-based axoneme which is wrapped into the ciliary membrane. The specific molecular composition of the cilium is maintained by the transition zone, a protein complex at the base of the PC (Malicki and Johnson, 2017) which functions as a gating region, regulating the trafficking of molecules and compartmentalizing this organelle. Molecules move bidirectionally across the cilium thanks to the intraflagellar transport (IFT) machinery: anterograde transport is mediated by the IFT-B complex bound to kinesin motor proteins (heterotrimeric kinesin 2), while retrograde transport is performed by IFT-A complex together with cytoplasmic dynein-2. These processes ensure ciliary functioning and homeostasis. As a primary signaling hub, the PC plays a key role during neurodevelopmental processes, indeed dysregulation of proteins involved in PC dynamics (i.e., basal bodies associated proteins) and signaling (i.e., AC3, INPP5E, SSTR3) have been linked to several neuronal phenotypes. In particular, PC is fundamental for Shh signaling, as the compartmentalized nature of this organelle enables the spatial organization of receptors and signaling mediators (Bangs and Anderson, 2017). The PC has been proposed to act also as a molecular switch regulating the balance between canonical and non-canonical Wnt pathways. Indeed, defective PC can alter the balance of canonical versus non-canonical Wnt signaling with context-dependent changes in β-catenin localization and activity (Guemez-Gamboa et al., 2014; Anvarian et al., 2019). Defects in cilia function, caused by altered function of proteins involved in PC dynamics and signaling, can therefore affect neural tube patterning, a process mainly regulated by morphogens gradient and respective signaling (Besse et al., 2011). Alterations of PC can also affect corticogenesis, in particular apical radial glia (aRG) proliferation, impacting symmetric and asymmetric division, differential neuronal migration routes, synapses formation and axon pathfinding (Higginbotham et al., 2012; Guadiana et al., 2013; Lepanto et al., 2016; Guo et al., 2019; Matsumoto et al., 2019; Hasenpusch-Theil et al., 2020; Hasenpusch-Theil and Theil, 2021; Meka et al., 2020; Toro-Tapia and Das, 2020; Tereshko et al., 2021; Thomsen et al., 2025). At the macroscopic level, these cellular alterations reverberate into microcephaly, macrocephaly, cerebellar hypoplasia, heterotopia, hydrocephalus phenotypes and neurobehavioral defects (Guo et al., 2015; Laclef et al., 2015; Malicki and Johnson, 2017; Youn and Han, 2018; Hasenpusch-Theil and Theil, 2021; Duy et al., 2022), associated with classical ciliopathies (i.e. Joubert syndrome) (Mitchison and Valente, 2017) but also to neurodevelopmental conditions with alterations in Hedgehog (Hh) pathway (especially Gli3/Gli3R modulation) (Hasenpusch-Theil and Theil, 2021; Shimada and Kato, 2022), Wnt/PCP and mTOR (Coschiera et al., 2024). Thus, during embryonic neurodevelopment, deleterious variants in genes directly (ciliary proteins) or indirectly (proteins modulating microtubules, centrosome localization and mitotic spindle dynamics) involved in ciliary functionality may associate to ASD conditions: a connection that warrants further investigation. In this mini-review, we synthesize the current knowledge on the genetics of ASD focusing on a selected group of ASD-related genes with strong associations to PC biology. We explore how these genes contribute to cellular processes including ciliary framework and highlight those with the strongest involvement in ciliary structure or signaling.

## 2 The intersection between ASD and primary cilium: a genes’ set enrichment analysis

To assess whether the overlap between ASD-related genes and ciliary genes exceeds random expectation, we performed an enrichment-based analysis starting from a curated list of 1238 ASD-related genes derived from SFARI Gene database (Q1 2026 release; May 1, 2026)^1^. SFARI Gene classifies genes into evidence-based categories (Scores 1–4 and Syndromic) according to the strength of the available genetic evidence from human studies. To provide a comprehensive overview of ASD genetics, all genes included in the Q1 2026 release (accessed 4/05/2026) were considered irrespective of their evidence score. We then generated the ciliary reference dataset by combining two complementary resources: the SYSCILIA Gold Standard version 2 (SysCilia GSv2), which is a manually curated collection of 686 experimentally validated ciliary genes, established by the SYSCILIA Consortium to define a high-confidence reference set (Vasquez et al., 2021). To increase coverage while maintaining high confidence, SysCilia GSv2 was complemented with CiliaCarta version 1 (van Dam et al., 2019; 935 unique genes), which instead uses naive Bayesian integration to combine multiple independent sources of evidence including proteomic, transcriptomic, evolutionary, protein–protein interaction and subcellular localization dataset. For each dataset, likelihood ratios comparing the probability of observing a given feature among known ciliary versus non-ciliary genes were estimated and combined according to Bayes’ theorem to calculate posterior odds of ciliary association. These posterior probabilities were transformed into a log-likelihood–based CiliaCarta Score, with positive values indicating increasing support for ciliary function and negative scores indicating progressively weaker support. High-confidence candidate genes were retained using a corrected false discovery rate (cFDR) threshold of ≤25%. Thus, while SYSCILIA v2 maximizes specificity through literature curation, CiliaCarta increases sensitivity by statistically prioritizing candidate ciliary genes from complementary large-scale datasets. Gene identifiers from both resources were harmonized according to the HGNC-approved gene symbols and duplicate entries were removed, yielding a merged reference dataset comprising 1182 unique ciliary genes. Overlapping genes were identified by exact matching of HGNC-approved symbols between the ASD-associated and the ciliary dataset, resulting in a final set of 105 shared genes, hereafter referred to as the CiliASD dataset (Figures 1A–C). Considering the human protein-coding genome as defined by GENCODE v49 (19433 protein-coding genes) and published datasets of ciliary gene content as described above, the expected overlap if our observation were solely due to chance between 1238 SFARI ASD-related genes and 1182 ciliary-associated genes would have been 75.3 ± 8.1 genes (mean ± SD under the null model). In contrast, we observed 105 overlapping genes, corresponding to a 1.39-fold enrichment (*z* = 3.65). Fisher’s exact test confirmed a significant enrichment (OR = 1.47, 95% CI = 1.18–1.82, two-tailed *p* = 4.5 × 10^–4^), permutation analysis yielded a consistent empirical *p*-value (*p* = 3.19 × 10^–4^) (Figures 1D–E). While not proving a direct causal relationship, these results show a statistically significant enrichment of ciliary-associated genes among ASD-related genes, supporting ciliary dysfunction as a potential convergent mechanism. Early evidence linking ASD to PC dysfunction emerged by the recognition that key ASD-relevant signaling pathways (i.e., Shh, Wnt, and mTOR) are critically dependent on intact PC. Subsequent identification of ciliary localization or function of multiple ASD risk genes consolidated the concept of ciliary dysfunction as a contributor to ASD pathogenesis. On the other hand, the study of syndromic ciliopathies with neurodevelopmental and ASD-like features, further strengthened this association (Reiter and Leroux, 2017). Specifically, functional classification of CiliASD genes (Figures 1E, 2A,B) revealed a strong convergence on biological processes related to ciliary structure and signaling, intracellular trafficking and neurodevelopment. A substantial fraction of CiliASD genes clustered into categories directly involved in ciliogenesis and basal body organization, including canonical ciliopathy genes such as *AHI1, BBS4, CEP290, OFD1* (Shaheen et al., 2016) and transcriptional regulators of ciliary gene expression as *RFX3-4-7* (Magnani et al., 2015). In parallel, a prominent group of genes encoded axonemal dyneins, kinesins and microtubule-associated proteins, underscoring the importance of microtubule-based transport and cytoskeletal dynamics shared between PC and developing neurons (Hirokawa et al., 2009; Ishikawa and Marshall, 2011). Notably, multiple ASD-associated genes mapped to vesicle trafficking and exocyst complexes (Winkler et al., 2025), highlighting the role of membrane trafficking in targeting receptors and signaling components to the cilium and synapse. Cilia-dependent signaling pathways, including cAMP, GPCR, Shh, mTOR and Notch signaling were recurrently represented, linking extracellular signal sensing at the PC to downstream pathways known to regulate neuronal proliferation, differentiation and synaptic maturation. Finally, several genes traditionally classified as synaptic or ion channels also exhibited documented ciliary localization or function, reinforcing the concept that ciliary dysfunction and synaptic alterations are not independent mechanisms but converge during corticogenesis and circuit formation (Guemez-Gamboa et al., 2014). Together, this functional stratification supports a model in which perturbation of ciliary structure, transport and signaling constitutes a unifying biological axis underlying a subset of ASD-associated genes.

**FIGURE 1:**
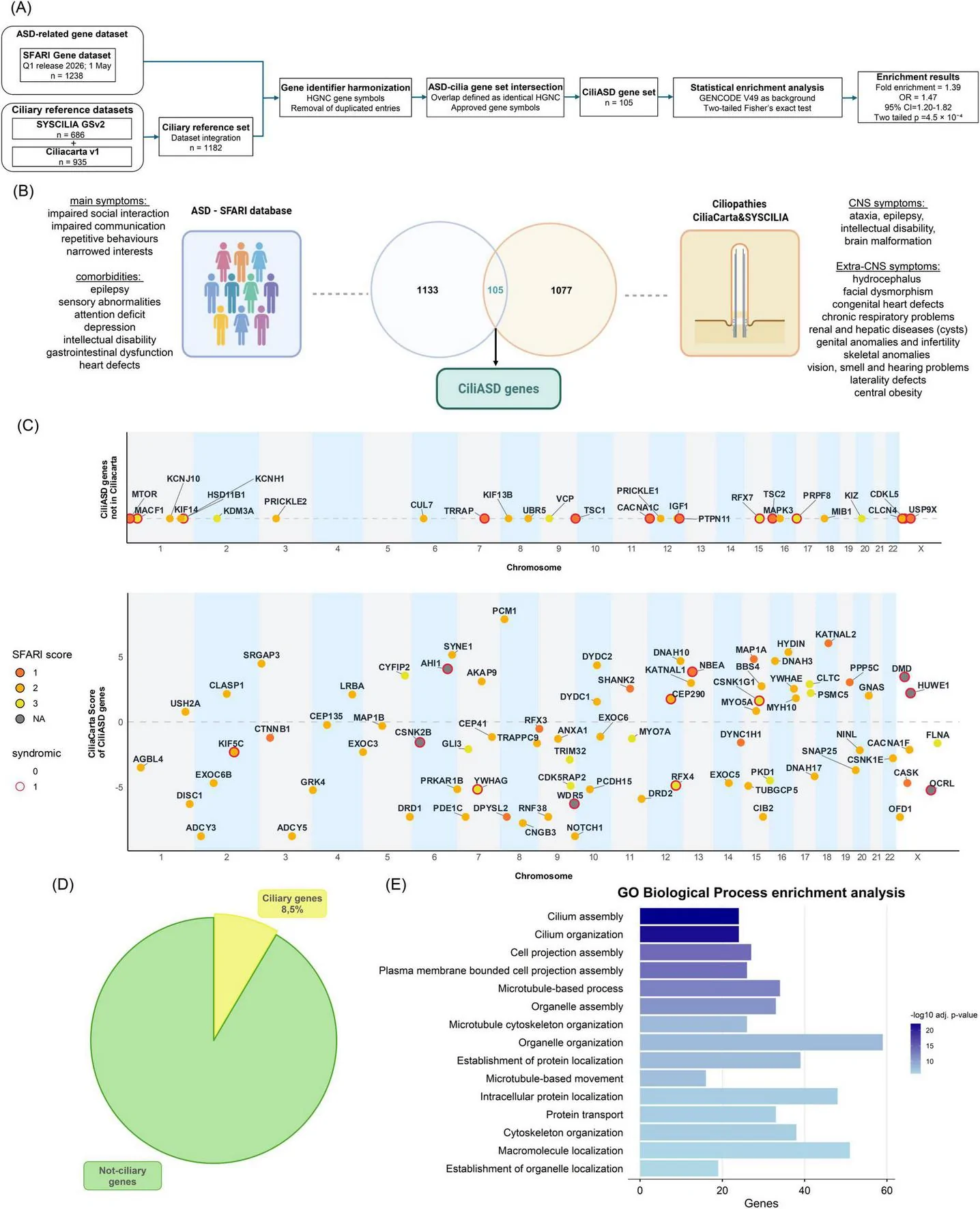
Identification and characterization of ASD-associated ciliary genes. **(A)** Flowchart for the identification of CiliASD genes: ASD-associated genes from the SFARI Gene database were harmonized and intersected with curated ciliary gene reference sets based on HGNC-approved gene symbols. **(B)** Overview of the intersection between ASD-associated genes from the SFARI Gene database and ciliary genes from CiliaCarta and SysCilia GSv2, identifying 105 shared genes defined as CiliASD dataset. Representative ASD and ciliopathy related clinical manifestations are also reported. **(C)** Manhattan plot in which dots indicate CiliASD genes, the *x*-axis indicates the genomic position (chromosomes 1-22 and X-chromosome) and the color indicated their relative SFARI score, according to the legend. On the bottom graph, *y* axis shows assigned CiliaCarta score of indicated CiliASD genes. The top graph, instead, shows genes absent from CiliaCarta, therefore no score is indicated. **(D)** Percentage of ciliary genes among the SFARI ASD-associated gene set, corresponding to 8.5% of the total annotations on SFARI base (Q1 2026 release; May 1, 2026). **(E)** Functional classification of CiliASD genes according to Gene Ontology categories. The number of genes for each class is indicated in the *x* axis. Adj. *p*-value is shown in different colors according to the legend.

**FIGURE 2:**
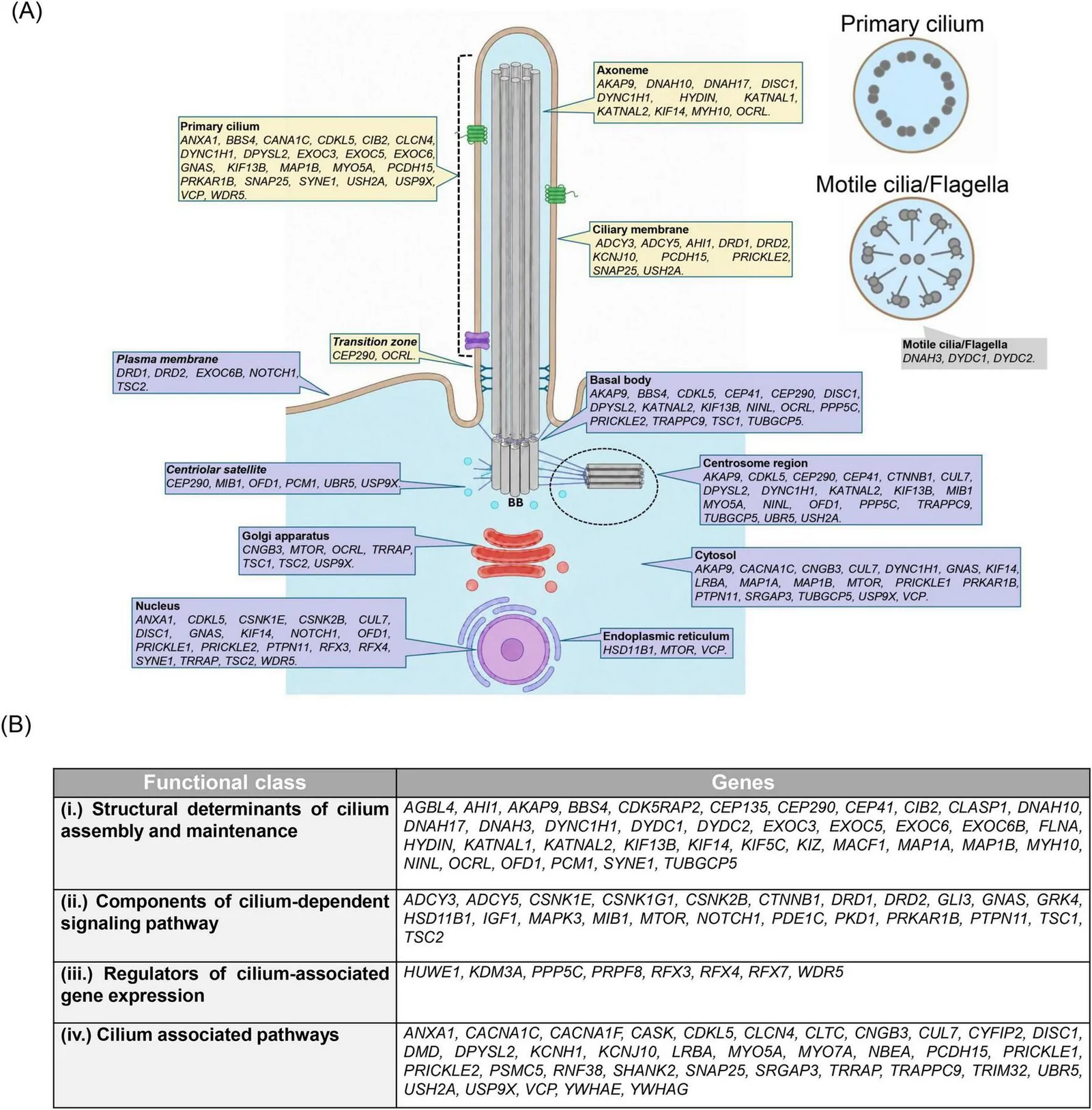
Ciliary localization of ASD-associated SFARI genes. **(A)** Schematic representation of primary cilium showing the distribution of CiliASD genes across cellular and ciliary compartments. Only genes with SFARI scores 1 and 2 are reported. Genes are grouped according to their documented localization. The insert on the right shows the structural organization of primary and motile cilia and lists genes associated with motile cilia/flagella. **(B)** Table listing the 105 genes of the CiliASD gene set. This gene set converges on four partially overlapping mechanistic modules, with a strong enrichment for (i) structural determinants of cilium assembly and maintenance, complemented by (ii) components of cilium-dependent signaling pathways, a smaller but conceptually important group of (iii) transcriptional regulators of ciliogenesis, and (iv). a subset of diverse pathways associated with the primary cilium.

## 3 Genetic underpinning of CiliASD genes

While most ASD-associated genes in CiliASD dataset follow a *de novo* dominant inheritance, a substantial subset (approximately one quarter of the dataset) shows recessive or X-linked inheritance, often corresponding to hypomorphic or dosage-sensitive perturbations of ciliary and centrosomal functions. This observation supports a model for which hypomorphic alleles of genes classically associated with recessive ciliopathies could associate to milder, non-syndromic phenotypes, including ASD. Such allelic distribution suggest a quantitative relationship between ciliary dysfunction and neurodevelopmental outcomes (Reiter and Leroux, 2017). Several genes in CiliASD dataset illustrate how quantitative perturbations of ciliary function may contribute to neurodevelopmental phenotypes relevant to ASD, even when classical ciliopathy features are attenuated. For instance, *CEP290* (Bachmann-Gagescu et al., 2015) and *AHI1* (Alvarez Retuerto et al., 2008) are well-established causes of recessive, syndromic ciliopathies when biallelic loss-of-function variants are present, with ASD and related neurodevelopmental features frequently reported as part of the syndromic spectrum. Importantly, hypomorphic variants or allelic combinations retaining partial residual function have been associated with milder neurodevelopmental presentations, in some cases including ASD or ASD-related traits, in the absence of overt multisystem involvement (Coppieters et al., 2010; Kilander et al., 2018). These observations support the concept of an allelic continuum, in which the degree of functional impairment of ciliary proteins, rather than the affected gene *per se*, shapes the severity and specificity of neurodevelopmental outcomes. A similar dosage-sensitive effect is suggested for *OFD1*, an X-linked gene whose complete loss leads to severe developmental syndromes (Chen et al., 2025), whereas milder or mosaic perturbations have been associated with isolated neurodevelopmental phenotypes (Poduri et al., 2013; Freed and Pevsner, 2016; Tran et al., 2020). This indicates that precise regulation of basal body and centrosomal components is critical for neurodevelopment and may be particularly vulnerable to functional disruption. In contrast, genes such as *KATNAL2*, which encodes a microtubule-severing enzyme involved in ciliary and centrosomal dynamics, are predominantly affected by *de novo* heterozygous variants in ASD cohorts. This pattern suggests that even modest alterations in microtubule remodeling can have disproportionate effects on cilium-dependent processes during brain development (O’Roak et al., 2012). Finally, transcriptional regulators of ciliogenesis, including *RFX3*, provide an additional layer of convergence. Rather than encoding structural components of the cilium, these factors orchestrate ciliary gene expression programs, and their disruption may indirectly impair multiple aspects of ciliary assembly and signaling (Choksi et al., 2014). Together, these examples underscore that ASD-associated variation converges not on a single ciliary defect, but on a spectrum of dosage-sensitive perturbations affecting ciliary structure, signaling and gene regulation.

## 4 CiliASD functional subgrouping

### 4.1 Ciliary structure, biogenesis and basal body compartment

A significant fraction of ASD-associated genes converges on molecular mechanisms governing PC assembly, basal body organization and centrosome function (Figures 1E, 2A,B). These genes encode core structural or regulatory components of the cilium-centrosome axis, a cellular hub increasingly recognized as critical for early human corticogenesis and neurodevelopmental disease (Reiter and Leroux, 2017; Breslow and Holland, 2019). In neural progenitor cells, the PC acts as a key signaling antenna integrating Shh, Wnt and Notch pathways, which collectively regulate progenitor proliferation, cell cycle exit and neuronal fate specification (Goetz and Anderson, 2010). Pathogenic variants in canonical ciliopathy genes such as *AHI1*, *CEP290*, *BBS4* and *OFD1* impair transition zone integrity and ciliary trafficking, leading to altered Shh signal transduction and aberrant cortical progenitor dynamics (Guemez-Gamboa et al., 2014; Park et al., 2019). Notably, altered Shh signaling has been directly implicated in ASD pathogenesis through its effects on cortical patterning, excitatory–inhibitory balance and synaptic circuit formation (Youn and Han, 2018). Several genes in CiliASD dataset further link centrosome biology and neurodevelopmental disorders: microtubule-severing enzymes as KATNAL1, KATNAL2 and kinesins (KIF13B, KIF14) regulate axonemal microtubule dynamics and centrosome-associated processes, thereby influencing mitotic spindle orientation and the balance between symmetric and asymmetric divisions of radial glial cells (Toro-Tapia and Das, 2020). Perturbations of these mechanisms have been shown to result in abnormal cortical thickness, disrupted laminar organization and neurodevelopmental phenotypes overlapping with ASD (Pilaz et al., 2016a,b). Centrosomal scaffold proteins such as PCM1 and TUBGCP5 further underscore the functional coupling between centrosome integrity and cilium formation. Variants in centrosomal genes have been associated with altered neurogenesis and increased ASD risk, supporting the hypothesis that centrosome–cilium uncoupling represents a shared pathogenic mechanism across genetically heterogeneous ASD cases (Insolera et al., 2014; Cavaco and Carvalhal, 2025). Collectively, these observations support a model in which ASD-associated genetic variation converges on the cilium–centrosome axis to perturb early corticogenesis, affecting neural progenitor behavior and cortical circuit assembly. This convergence provides a mechanistic framework linking ciliopathy-related pathways to ASD susceptibility and suggests that subtle defects in ciliary signaling, rather than overt ciliopathy phenotypes, may underlie a substantial subset of ASD cases.

### 4.2 Microtubule-based transport, vesicle trafficking and cilia-dependent signaling

A widely represented group of ASD-associated genes in CiliASD dataset converges on axonemal dyneins, microtubule-based transport, vesicle trafficking and cilia-dependent intracellular signaling, highlighting the functional integration of ciliary structure with neuronal signaling and cortical circuit assembly (Figures 1E, 2A,B). Axonemal dyneins and dynein-associated components (DNAH3, DNAH10, DNAH17, DYDC1, DYDC2) together with cytoplasmic motors and cytoskeletal regulators (CLASP1, DYNC1H1, FLNA, KIF5C, MACF1,MAP1A, MAP1B, MYH10) are essential for microtubule dynamics, intracellular transport and neuronal polarization. Disruption of these processes has been shown to impair neuronal migration, axon elongation and synaptic organization, core developmental features frequently altered in ASD (Franker and Hoogenraad, 2013). As an example, *DYNC1H1* pathogenic variants are associated with neurodevelopmental syndromes featuring ASD traits, linking defective dynein-mediated transport to altered corticogenesis and connectivity (Willemsen et al., 2012; Hoang et al., 2017). In parallel, multiple ASD genes in this set encode components of vesicle trafficking and endomembrane systems (CLTC, EXOC3, EXOC5, EXOC6, EXOC6B, LRBA, OCRL, PSMC5, TRAPPC9, UBR5, VCP), processes that are tightly coupled to ciliogenesis, receptor recycling and synaptic function. The exocyst and TRAPP complexes are required for targeted membrane delivery to the base of the cilium and for polarized trafficking in neurons, while clathrin and ubiquitin dependent pathways regulate turnover of signaling receptors and synaptic proteins (Das and Guo, 2011; Nechipurenko et al., 2013). Perturbation of these pathways has been implicated in ASD through effects on synapse maturation, neuronal homeostasis and ciliary signaling competence. Crucially, several CiliASD genes directly modulate cilia-dependent signaling cascades central to cortical development including cAMP (Paolocci and Zaccolo, 2023), Shh, Wnt, mTOR and Notch pathways (*ADCY3, ADCY5, CSNK1E, CSNK1G1, CSNK2B, DRD1, DRD2, GRK4, GNAS, GLI3, IGF1, MTOR, NOTCH1, PKD1, PRKAR1B, PPP5C, PTPN11, TSC1, TSC2*). Primary cilia act as hubs for these pathways in neural progenitors, regulating proliferation, fate specification and excitatory-inhibitory balance. Dysregulation of ciliary Shh and mTOR signaling in particular, has been repeatedly linked to ASD-related phenotypes, including altered cortical thickness, synaptic dysfunction and abnormal network activity (Youn and Han, 2018; Park et al., 2019). Together, these observations support a convergent model in which ASD risk genes impact neuronal development by disrupting cilia-dependent transport and signaling networks, thereby coupling defects in intracellular trafficking and cytoskeletal dynamics to altered corticogenesis and ASD susceptibility (Guo et al., 2017).

### 4.3 Regulators of cilium-associated gene expression

Regulation of cilium-associated gene expression is orchestrated by a network of transcription factors and epigenetic regulators, including members of the RFX family (RFX3, RFX4, RFX7) and chromatin modifiers (KDM3A, WDR5). RFX transcription factors act as master regulators of the ciliogenic program coordinating the expression of structural and functional components of the PC (Sugiaman-Trapman et al., 2018; Coyle et al., 2023), implicated in dysregulation of ciliary gene expression in ASD (Lai et al., 2025). In parallel, several ASD risk genes encode chromatin modifiers and transcriptional regulators (*KDM3A*, *MIB1, RNF38, WDR5*) that control histone modifications (Yao et al., 2016), enhancer activity and Notch-dependent transcriptional programs. Disruption of these factors has been linked to altered neurogenesis, cortical patterning defects and ASD-related phenotypes (Bjornsson et al., 2014; De Rubeis and Buxbaum, 2015; Satterstrom et al., 2020). Importantly, epigenetic regulators such as WDR5, are increasingly recognized as hubs integrating developmental signaling pathways that are spatially organized at the PC during corticogenesis, thereby linking chromatin regulation to cilia-mediated signaling in ASD (Shi et al., 2014; Kulkarni et al., 2018).

### 4.4 Ion channels and neuronal excitability, neuronal migration and synaptic functions

Another subset of CiliASD genes converges on mechanisms regulating neuronal excitability, migration, polarity and synaptic function, highlighting how early developmental processes and mature circuit function are mechanistically intertwined in ASD (Figures 1E, 2A,B). Genes encoding ion channels and regulators of membrane excitability (*CACNA1C*, *CACNA1F*, *CLCN4*, *CNGB3*, *KCNH1*, *KCNJ10*) play essential roles in controlling neuronal firing, calcium signaling and activity-dependent gene expression (Jin et al., 2014; Muntean et al., 2014; Nakagawa-Tamagawa et al., 2021; Rosenberg and Spitzer, 2011; Bading, 2013; Schneggenburger and Rosenmund, 2015; Napoli et al., 2022). Disruption of these channels has been repeatedly associated with ASD (Liao and Li, 2020; Kessi et al., 2021) and related neurodevelopmental phenotypes, as exemplified by *CACNA1C* pathogenic variants in Timothy syndrome (Splawski et al., 2004) and *CLCN4* variants in X-linked intellectual disability with autistic features (Palmer et al., 2018). Importantly, emerging evidence indicates that ion channel activity is integrated with ciliary signaling networks during corticogenesis, where regulation of Ca^2 +^ contributes to the control of progenitor cell cycle progression and neuronal differentiation (Guemez-Gamboa et al., 2014; Saternos et al., 2020; Shim et al., 2023; Vien et al., 2023; Sanchez et al., 2025). In parallel, several ASD risk genes regulate neuronal radial migration of excitatory neurons, planar cell polarity, cytoskeletal remodeling and cortical layering (*CDK5RAP2*, *CTNNB1, CYFIP2, DISC1*, *DPYSL2*, *PRICKLE1*, *PRICKLE2*, *SRGAP3*, *SYNE1*) (Marley and von Zastrow, 2010; Wang and Brandon, 2011; Desprez et al., 2023; Song et al., 2026). These processes are indeed highly sensitive to disruptions in centrosome positioning and microtubule dynamics (Higginbotham and Gleeson, 2007; Tissir and Goffinet, 2010; Valiente and Marín, 2010; Buchsbaum and Cappello, 2019; Smith et al., 2020). Perturbations in these pathways lead to altered cortical architecture, abnormal connectivity and behavioral phenotypes overlapping with ASD (Ishizuka et al., 2011; Stoner et al., 2014). Notably, several of these factors intersect with Wnt and PCP signaling, which are modulated at the PC and are critical for cortical patterning (Boitard et al., 2015; Youn and Han, 2018). Finally, a core group of ASD-associated genes encodes synaptic proteins central to neurotransmitter release, synaptic scaffolding and activity-dependent plasticity (CASK, CDKL5, NBEA, SHANK2, SNAP25, YWHAE, YWHAG) (Südhof, 2008; Bourgeron, 2015; Chen et al., 2021; Volos et al., 2025). Disruption in these genes results in impaired synapse formation, altered excitatory-inhibitory balance and network dysfunction (Nelson and Valakh, 2015; Monteiro and Feng, 2017). Increasing evidence suggests that synaptic defects in ASD often arise downstream of earlier neurodevelopmental perturbations, including those affecting ciliary signaling, neuronal migration and excitability (State and Šestan, 2012; Willsey et al., 2013; Guemez-Gamboa et al., 2014).

### 4.5 Miscellaneous group

A distinct but mechanistically related subset of CiliASD genes is classically associated with sensory ciliopathies affecting the retina and inner ear (*CIB2*, *CNGB3*, *KIZ*, *MYO7A*, *PCDH15*, *USH2A*). Although originally described in syndromic forms of blindness and deafness, pathogenic variants in these genes are increasingly reported in individuals with ASD, often accompanied by sensory processing abnormalities, a core feature of autism (Marco et al., 2011; Jurisch-Yaksi et al., 2024). These proteins localize to specialized PC or cilia-derived structures in photoreceptors and hair cells, where they regulate mechano-transduction, calcium signaling and cytoskeletal coupling. Growing evidence suggests that similar cilia-dependent sensory signaling mechanisms operate in developing cortical neurons, influencing activity-dependent circuit refinement and social behavior (Jurisch-Yaksi et al., 2024). Finally, several ASD-associated genes traditionally linked to muscle or cytoskeletal function (*ANXA1*, *CUL7*, *DMD*, *HSD11B1*, *MYO5A*, *TRIM32*) play broader roles in neuronal morphology, intracellular trafficking and mechano-transduction. Beyond their canonical functions, these genes regulate actin–microtubule interactions, vesicle transport and stress-responsive signaling pathways essential for neuronal migration and synaptic stability. Notably, *DMD* and *MYO5A* variants are associated with neurodevelopmental and behavioral phenotypes independent of overt muscle pathology, highlighting a direct contribution to brain development.

## 5 Extra-CNS phenotypes of CiliASD gene subset

Autism spectrum disorder is frequently associated with several comorbidities, including ADHD, anxiety, depression, epilepsy, intellectual disability, gastrointestinal dysfunction and sensory abnormalities. Increasing evidence suggests a partial overlap between ASD and ciliopathies not only in neurodevelopmental phenotypes, but also in extra-CNS manifestations. Notably, approximately one third of the ASD genes identified in CiliASD dataset have been independently linked to congenital malformations affecting the heart, skeletal system or genitourinary tract, consistent with the known role of PC in embryonic patterning and organogenesis. Indeed, many of these defects are often caused by the dysfunction of cilia mediated signaling (especially GPCRs, TGF-β/BMP, as well as the aforementioned Shh and Wnt) in specific cell types and during precise developmental stages, supporting the hypothesis that altered cilia dynamics could represent a common etiology for both CNS and extra-CNS features in ASD comorbidities and ciliopathies.

### 5.1 Comorbidity phenotypes in ASD and in ciliopathies

Alterations in cilia components shared between primary and motile cilia can result in malfunction of specialized cilia that are present in sensory organs and responsible for vision, smell and hearing. Photoreception in cone and rod cells is mediated by GPCRs signaling and when this process is disrupted, either due to detrimental variants or because of cilia structural defects (like IFT components or *Kif3a* variants) (Fliegauf et al., 2007), it causes retinal degeneration through apoptosis, possibly because of defective opsin trafficking (Reiter and Leroux, 2017). This could explain why *CACNA1F, CDK5RAP2, CEP290, CTNNB1, CIB2, MYOA5A, OCRL, PCDH15, PSMC5* variants can result in different eye defects, including blindness due to Leber congenital amaurosis and retinitis pigmentosa. Different ciliopathies present with deafness, which might instead be caused by functional impairment of the kinocilium present in the acoustic-vestibular cells, responsible for the transduction of sound vibration (Moon et al., 2020). This phenotype is present in individuals with variants in *CIB2, MAP1B, MYO7A, PCDH15, IGF1, KCNH1, KCNJ10, KIF13B*.

### 5.2 Extra-CNS phenotypes typical of canonical ciliopathies

Several phenotypes are more characteristic of classical ciliopathies but still overlap with ASD-associated syndromic conditions. These include congenital heart defects (CHDs), craniofacial dysmorphisms and skeletal abnormalities. A series of heart problems are present in the phenotypic spectrum of ciliopathies (Djenoune et al., 2022) and there are several evidence of the importance of ciliary function in organ and cardiac development (Anvarian et al., 2019). Cilia disruption can therefore result in congenital heart defects. Interestingly, some of the genes from CiliASD lead to CHD when associated with pathogenic variants, as *FLNA*, *MYH10*, *PSMC5*, *WDR5*, *MIB1*, *TSC1*. Notably, a link between ASD, CHD and PC was also investigated by Teerikorpi et al. (2025). Facial dysmorphisms are typical facial features that are present in about 30% of ciliopathies (Brugmann et al., 2010; Moore, 2022). They develop as a consequence of altered craniofacial development and they might be caused by defective neural crest cell migration (Tobin et al., 2008). As well as by malfunction of signaling pathways that participate in craniofacial development, whose signaling is mediated or modulated by the PC (Brugmann et al., 2010; Zaghloul and Brugmann, 2011). A part of the genes selected from CiliASD datasets, when harboring pathogenic variants, result in syndromes with distinct features, including facial dysmorphisms. The pathways regulating craniofacial development are also involved in skeletal development and skeletal abnormalities are also often present in the clinical spectrum of ciliopathies (Hwang et al., 2018; Butterfield et al., 2009; Handa et al., 2020; Reiter and Leroux, 2017). Among CiliASD dataset, the pathogenic variants of several genes result in skeletal phenotypes, often in combination with other clinical features (*CASK, CLTC, CSNK2B, DYNC1H1, GNAS, OFD-1, WDR5, NINL, SYNE1, CUL7, CLCN4, RFX7*). Altogether, these observations support the hypothesis that altered cilia structure, dynamics and signaling may represent a common pathogenic mechanism underlying both neurodevelopmental and systemic phenotypes observed in ASD and ciliopathies.

## 6 Discussion and conclusions: limitations and open questions

The molecular basis of ASD remains highly heterogeneous and a major challenge is to identify biological mechanisms that converge across diverse genetic etiologies. In this study, we identified the CiliASD gene set, revealing a significant enrichment of ASD-associated genes within ciliary-related compartment and pathways. Rather than suggesting a universal mechanism, these findings support the existence of a biologically defined subset of ASD in which alterations of ciliary structure, trafficking and signaling may contribute to neurodevelopmental dysfunction. The enrichment observed across curated ciliary resources supports previous observations linking individual ASD-associated genes to ciliogenesis, ciliary signaling and centrosome-related functions. Importantly, the integration of experimentally supported ciliary genes with broader computationally prioritized candidates allowed the identification of both established and emerging cilia-associated ASD genes, highlighting shared molecular modules. However, several limitations should be considered. First, the strength of evidence supporting ciliary involvement differs among genes. While genes such as *CEP290*, *OFD1*, *KATNAL2*, and *RFX3* have experimentally validated roles in ciliogenesis or cilia-dependent signaling, other genes within the CiliASD dataset are supported mainly by computational predictions, protein–protein interaction networks or subcellular localization annotations. These candidates therefore require functional validation, particularly in neurodevelopmentally relevant cellular models. Second, available experimental evidence largely derives from cellular systems or model organisms and may not fully reflect the complexity of human brain development. In particular, ASD-associated variants frequently involve partial functional alterations, including heterozygous or hypomorphic effects, which may generate subtle changes in ciliary signaling distinct from the severe loss-of-function variants typically associated with classical ciliopathies. Understanding how these dosage-sensitive perturbations influence neuronal development remains an important challenge. Third, the identification of a ciliary role does not imply that ciliary dysfunction represents the sole pathogenic mechanism for all ASD-associated variants. Many ciliary proteins also regulate extraciliary processes, including centrosome biology, microtubule dynamics, intracellular trafficking and transcriptional regulation. Therefore, ASD phenotypes may arise from the combined disruption of ciliary and non-ciliary functions in a context-dependent manner. In conclusion, this study identifies a significant convergence between ASD-associated genes and ciliary biology, defining the CiliASD gene set as a framework to investigate emerging cilia-related mechanisms in neurodevelopment. While ciliary dysfunction is unlikely to represent a universal cause of ASD, our findings suggest that alterations in ciliary signaling and organization may contribute to disease risk in a molecularly defined subset of cases. Future studies integrating human genomic data with quantitative cellular phenotyping and *in vivo* models will be required to determine the developmental timing, molecular specificity, and clinical relevance of cilia-related mechanisms in ASD. This may provide a new perspective for dissecting ASD heterogeneity and may facilitate the identification of novel genotype–phenotype relationships and therapeutic opportunities.

## Footnotes

1
https://gene.sfari.org/database/human-gene
